# Editorial: Aging: challenges and opportunities for inclusion and active participation, volume II

**DOI:** 10.3389/fpsyg.2026.1938855

**Published:** 2026-08-18

**Authors:** Chiara Aleffi, Ramona Bongelli, Alessandra Fermani, Paola Nicolini, Veronica Guardabassi

**Affiliations:** 1 Department of Education, Cultural Heritage and Tourism, University of Macerata, Macerata, Italy; 2 Department of Political Science, Communication and International Relations, University of Macerata, Macerata, Italy; 3 Department of Humanities – Languages, Language Liaison, History, Arts, Philosophy, University of Macerata, Macerata, Italy

**Keywords:** active aging, community participation, lifelong learning, resilience, social inclusion, wellbeing

## 1 Introduction

Population aging remains one of the defining demographic transformations of the 21st century, bringing unprecedented opportunities as well as important social, psychological, and healthcare challenges. Although increasing life expectancy reflects important improvements in health and living conditions, it also poses significant challenges for healthcare systems, social policies, and local communities. Aging is a heterogeneous process, characterized by substantial differences between individuals and influenced by biological, psychological, social, and environmental factors. For this reason, promoting active aging requires moving beyond the traditional focus on disease and disability, while creating opportunities for older adults to remain socially connected, engaged, and included in their communities.

Building upon the first volume of this Research Topic, *Aging: Challenges and Opportunities for Inclusion and Active Participation – Volume II* further explores the challenges and opportunities associated with inclusion and active participation in later life. The articles collected here address different aspects of aging from psychological, social, educational, communicative, and public health perspectives. Although they investigate heterogeneous topics, they all contribute to a better understanding of the factors that may promote wellbeing and participation among older adults in contemporary societies.

## 2 The Research Topic

The 12 articles included in this second volume reflect the growing complexity of contemporary aging research. Rather than focusing exclusively on health outcomes, they examine the multiple dimensions that enable older adults to remain active participants in society, highlighting the importance of inclusive environments capable of fostering wellbeing throughout later life.

Although diverse in their objectives and methodologies, the contributions can be broadly grouped into four complementary thematic areas: (1) community participation and social inclusion; (2) psychological wellbeing and social inequalities; (3) health promotion and quality of life; and (4) resilience, lifelong learning, and future engagement. Together, these studies reinforce the idea that successful aging should be understood as a dynamic process involving reciprocal interactions between individuals and the communities in which they live.

### 2.1 Community participation and social inclusion

The first thematic area includes studies highlighting the importance of social participation in promoting active aging. Different community settings and shared activities are presented as opportunities for strengthening interpersonal relationships, reducing loneliness, and fostering a greater sense of belonging.

Tan et al. explore the role of traditional tea houses as places where older adults can develop meaningful social relationships and experience emotional support within their communities. Similarly, Sun and Wu investigate older adults' satisfaction with community dining services, showing that these initiatives represent not only nutritional support but also valuable opportunities for social interaction.

Participation is also promoted through cultural and recreational activities, in particular traditional handicrafts may contribute to active aging by encouraging social participation and intergenerational exchange (Igreja et al.), while Guardabassi et al. show that board games represent a promising non-pharmacological intervention capable of enhancing wellbeing among older adults with dementia.

Collectively, these contributions underline the idea that social participation is not limited to formal programs or institutional interventions. Rather, it develops through accessible community spaces and meaningful shared experiences that allow older adults to remain active contributors to social life.

### 2.2 Psychological wellbeing and social inequalities

A second group of articles focuses on psychological wellbeing and on the social factors that may either facilitate or hinder successful aging.

Ageism emerges as a persistent challenge, with evidence suggesting that age-based discrimination negatively affects life satisfaction and disproportionately impacts older adults living in disadvantaged contexts (Guo and Li). Likewise, the growing digitalization of contemporary societies presents both opportunities and risks. While digital technologies have the potential to enhance participation and access to resources, the findings reported by Feng et al. reveal that these benefits depend on digital literacy, confidence, and supportive social environments. Simply providing technological access is insufficient if older adults continue to experience exclusion from digital participation.

The transition to retirement also illustrates the importance of psychosocial resources. Zhang et al. show that intentions to re-engage in work are shaped not only by personal resources but also by trust in others and by perceptions of social support, confirming that individual decisions are deeply embedded within broader relational contexts.

Overall, these studies suggest that psychological wellbeing cannot be understood independently from the broader social context. Inclusive environments, supportive relationships, and equal opportunities remain fundamental conditions for promoting active participation among older adults.

### 2.3 Health promotion and quality of life

Health promotion represents another important theme emerging from this Research Topic and the articles integrate physical, psychological, and social dimensions of wellbeing.

Li et al. examine the association between physical exercise and health-related quality of life, confirming the importance of maintaining active lifestyles throughout older adulthood. Similarly, Bai et al. investigate the effects of different exercise programs on physical fitness among middle-aged and older women, providing further evidence of the benefits of structured physical activity.

A broader perspective on health is offered by Simsek et al., who explore the relationship between voice outcomes following dental treatment and overall life satisfaction. Their findings highlight how health-related interventions may influence not only physical functioning but also communication, social relationships, and psychological wellbeing.

Together, these contributions reinforce the idea that healthy aging should be considered as a multidimensional process, in which physical, psychological, and social dimensions are closely interconnected.

### 2.4 Resilience, productive engagement, and lifelong learning

The final thematic area focuses on the future, emphasizing the continued potential for growth, adaptation, and contribution during older adulthood.

Positive expectations regarding aging are associated with greater productive engagement (Dai et al.), suggesting that beliefs about the aging process itself may influence opportunities for participation. At the same time, research involving older adult learners (Gao et al.) highlights the role of different forms of social support in strengthening resilience and sustaining lifelong learning.

These studies collectively challenge deficit-oriented representations of aging. Instead, they portray later life as a developmental phase characterized by ongoing learning, social contribution, and personal growth, provided that appropriate educational, social, and community resources remain available.

## 3 Conclusion

The articles included in *Aging: Challenges and Opportunities for Inclusion and Active Participation – Volume II* expand and consolidate the perspectives introduced in the first volume of this Research Topic. Across diverse disciplines and cultural settings, they consistently demonstrate that active aging emerges from the interaction between individual capabilities and enabling environments that foster participation, inclusion, health, and lifelong development.

At the same time, the contributions highlight the persistence of important challenges, including digital inequalities, and structural barriers that continue to restrict opportunities for many older adults. Addressing these issues requires interdisciplinary research capable of integrating psychological, educational, public health, and social perspectives, as well as evidence-informed policies that promote age-friendly communities.

This second volume reinforces a fundamental message: aging should not be understood simply as a process of adaptation to decline, but as a stage of life rich in opportunities for participation, resilience, learning, and meaningful contribution. This perspective resonates with Erikson's view of later life as the developmental stage in which individuals strive to achieve ego integrity rather than despair (Erikson and Erikson, 2013). Although the studies included in this Research Topic address different aspects of aging, they consistently suggest that supportive social environments, meaningful participation, and lifelong learning can foster a positive experience of later life and contribute to this developmental process. By bringing together complementary perspectives from different disciplines and countries, the Research Topic contributes to a growing body of evidence supporting more inclusive and sustainable approaches to aging in contemporary societies.
